# Performance of primary health care workers in detection of mental disorders comorbid with epilepsy in rural Ethiopia

**DOI:** 10.1186/s12875-021-01551-4

**Published:** 2021-10-14

**Authors:** Ruth Tsigebrhan, Abebaw Fekadu, Girmay Medhin, Charles R. Newton, Martin J. Prince, Charlotte Hanlon

**Affiliations:** 1 Centre for Innovative Drug Development and Therapeutic Trials for Africa (CDT-Africa), College of Health Sciences, Addis Ababa University, Addis Ababa, Ethiopia; 2 Department of Psychiatry, WHO Collaborating Centre in Mental Health Research and Capacity-Building, School of Medicine, College of Health Sciences, Addis Ababa University, Addis Ababa, Ethiopia; 3 Infection & Global Health Department, Brighton and Sussex Medical School, Brighton, UK; 4 Centre for Affective Disorders, Department of Psychological Medicine, Institute of Psychiatry, Psychology and Neuroscience, King’s College London, London, UK; 5 Aklilu-Lemma Institute of Pathobiology, Addis Ababa University, Addis Ababa, Ethiopia; 6 Department of Psychiatry, University of Oxford, Warneford Hospital, Warneford Lane, Oxford, UK; 7 Centre for Global Mental Health, Health Services and Population Research Department, Institute of Psychiatry, Psychology and Neuroscience, King’s College London, London, UK

**Keywords:** Epilepsy, Mental disorders, Detection, Africa, Primary health care

## Abstract

**Background:**

Timely detection and management of comorbid mental disorders in people with epilepsy is essential to improve outcomes. The objective of this study was to measure the performance of primary health care (PHC) workers in identifying comorbid mental disorders in people with epilepsy against a standardised reference diagnosis and a screening instrument in rural Ethiopia.

**Methods:**

People with active convulsive epilepsy were identified from the community, with confirmatory diagnosis by trained PHC workers. Documented diagnosis of comorbid mental disorders by PHC workers was extracted from clinical records. The standardized reference measure for diagnosing mental disorders was the Operational Criteria for Research (OPCRIT plus) administered by psychiatric nurses. The mental disorder screening scale (Self-Reporting Questionnaire; SRQ-20), was administered by lay data collectors. The sensitivity, specificity, positive predictive value (PPV) and negative predictive value (NPV) of PHC worker diagnosis against the reference standard diagnosis was calculated. Logistic regression was used to examine the factors associated with misdiagnosis of comorbid mental disorder by PHC workers.

**Results:**

A total of 237 people with epilepsy were evaluated. The prevalence of mental disorders with standardised reference diagnosis was 13.9% (95% confidence interval (CI) 9.6, 18.2%) and by PHC workers was 6.3% (95%CI 3.2, 9.4%). The prevalence of common mental disorder using SRQ-20 at optimum cut-off point (9 or above) was 41.5% (95% CI 35.2, 47.8%). The sensitivity and specificity of PHC workers diagnosis was 21.1 and 96.1%, respectively, compared to the standardised reference diagnosis. In those diagnosed with comorbid mental disorders by PHC workers, only 6 (40%) had SRQ-20 score of 9 or above. When a combination of both diagnostic methods (SRQ-20 score ≥ 9 and PHC diagnosis of depression) was compared with the standardised reference diagnosis of depression, sensitivity increased to 78.9% (95% (CI) 73.4, 84.4%) with specificity of 59.7% (95% CI 53.2, 66.2%). Only older age was significantly associated with misdiagnosis of comorbid mental disorders by PHC (adjusted odds ratio, 95% CI = 1.06, 1.02 to 1.11).

**Conclusion:**

Routine detection of co-morbid mental disorder in people with epilepsy was very low. Combining clinical judgement with use of a screening scale holds promise but needs further evaluation.

**Supplementary Information:**

The online version contains supplementary material available at 10.1186/s12875-021-01551-4.

## Background

There have been a number of studies indicating the high prevalence of comorbid mental disorders in people with epilepsy [1, 2]. The pooled prevalence of comorbid depression in people with epilepsy is estimated to be 22.9% (95% confidence interval (CI) 18.2–28.4%) and co-morbid anxiety disorders is 20.2% (95% CI 15.3–26.0%), based largely on studies from high-income countries (HIC) [2]. In hospital-based studies from sub-Saharan Africa, prevalence estimates for comorbid depression in people with epilepsy are even higher, e.g. 45.5% [3].

The relationship between epilepsy and mental disorders is complex [4]. There is an increased incidence (Incidence rate ratio (IRR) 1.5–15.7) of depression, anxiety and psychotic disorders before the onset of epilepsy compared to people who do not develop epilepsy [5, 6]. The incidence of these mental disorders is also increased (IRR 2.2–10.9) after the diagnosis of epilepsy compared to people with no epilepsy [5]. The complex causal interrelationship between depression and epilepsy has been most investigated [6]. Functional and structural neuroimaging studies provide evidence of common neuropathology underlying the association between depression and epilepsy [4, 6]. Regardless of the nature of the relationship, the existence of co-morbid mental disorders in people with epilepsy has a detrimental effect on prognosis of epilepsy and quality of life [4] .

Early detection and appropriate management of comorbid mental disorders in people with epilepsy is of paramount importance [4]. The evaluation should include lifetime and current history, family history of depressive, anxiety or attention deficit hyperactivity disorder (ADHD) and psychotic disorder. However, in routine clinical practice there is under-detection and limited treatment of comorbid depression and anxiety in people with epilepsy (PWE) [7]. Failure to recognize and properly manage these comorbid disorders has serious impacts [7]. Those who are diagnosed with mood disorders before the onset of epilepsy have been found to have an increased risk of treatment resistant epilepsy [8] and of developing adverse side effects of anti-epileptic medications [9]. The risk of suicide is also increased three-fold in people with epilepsy compared to the those without epilepsy [10].

In low- and lower middle-income countries (LLMICs), the treatment gap (PWE who are not accessing or unable to access biomedical facilities for diagnosis and treatment and, if accessing biomedical treatment, those not adhering to the prescribed anti-epileptic drug (AED)) for active epilepsy ranges from 25 to 100% [11, 12], even before considering access to care for co-morbid mental health conditions. The treatment gap for mental disorders in the general population in LLMICs is high, ranging from 1.6–15.4% [13]. In recognition of the high treatment gap for both epilepsy and mental disorders globally, the World Health Organization (WHO) recommends integrated management of priority mental, neurological and substance use disorders (MNS) at primary health care level [14, 15]. As part of this effort, the WHO has produced the mental health Gap Action Programme Intervention Guide (mhGAP-IG) comprising evidence-based clinical algorithms for MNS disorders that are suitable for non-specialist workers [14]. Epilepsy is included in mhGAP-IG, together with depression, psychosis, substance use disorders, suicidal behaviour, developmental disorders/child mental health problems, dementia and somatic/anxiety symptoms. Since its launch in 2008, there have been many efforts to adapt, implement and evaluate mhGAP, although there has been limited evaluation of the application of mhGAP-IG to people with co-morbid epilepsy and mental health disorders [16].

In Ethiopia, implementation of mhGAP in primary care settings has been shown to improve identification of people with psychosis and epilepsy [17], but not depression [18]. Qualitative exploration indicated unmet emotional needs in the people receiving mhGAP epilepsy care [19]. However, there have been very few studies to investigate the identification of comorbid mental disorders or the validation of common mental disorders screening instruments in people with epilepsy, especially in sub Saharan Africa [20]. The impact of using a locally validated screening tool on detection of comorbid mental disorders in people with epilepsy was found to improve detection in Zambia [21, 22]. However, further studies are needed to evaluate and determine the detection of comorbidity among people with epilepsy in primary care settings in order to quantify the treatment gap, and to inform the design of interventions to increase detection.

The aim of the current study was to evaluate the performance of primary health care (PHC) workers in detecting comorbid mental disorders, by comparing them to a screening scale and comparing their detection to a standardised reference diagnosis of comorbid mental disorders in people with epilepsy in rural Ethiopia. It also tries to clarify the misdiagnosis of PHC in relation to the different sociodemographic factors.

## Methods

### Study design

In this paper we assessed the diagnostic performance of trained PHC workers compared to both self-report symptom questionnaires and standardised clinical assessments using data collected in a cross-sectional study. This study was nested within the baseline of a cohort study to measure the impacts of comorbid mental disorders on quality of life, functioning and seizure control in people with epilepsy in rural Ethiopia.

### Setting

The study was conducted in four selected districts of the Gurage zone in Southern Nations, Nationalities and Peoples’ region (SNNPR) of Ethiopia. The zone is subdivided into 15 districts (*woredas*). The current study covered four of these 15 districts (Sodo, Eja, Wolikete and Kebena).

This study was part of the scale-up phase of the PRogramme for Improving Mental health CarE (PRIME) project [23]. PRIME was a research programme consortium across five low- and middle-income countries (Ethiopia, South Africa, Uganda, India and Nepal) that aimed to develop comprehensive evidence for the best strategy to integrate and scale up mental health care incorporated within primary health care settings. The PRIME-Ethiopia study focused on four priority MNS disorders, based on their associated disability and prioritisation by the Federal Ministry of Health of Ethiopia: psychosis, depression, epilepsy and alcohol use disorders. In PRIME, researchers worked with community stakeholders using a participatory process to develop a district mental health care plan [24, 25] which included delivery of mhGAP-IG in primary healthcare facilities, as well as interventions for community mobilisation and health system strengthening [24]. Health workers staffing primary healthcare facilities are health officers and nurses, but not physicians. PRIME had three phases: inception, implementation and scale up phases. Therefore, the PRIME project has major role in this study for the integration of mental health care in the primary health care facilities and training of the PHC workers in mhGAP-IG. One health centre from each district of the Gurage zone was selected as part of the scale up phase of PRIME [26].

In this study we included scale-up health centres of the PRIME project which were accessible and where there were sufficient numbers of people with epilepsy attending (*n* = 3 health centres). We additionally included health centres from the PRIME implementation site (*n* = 8 facilities) in the district of Sodo.

#### Sample size

The sample size was calculated for the cohort study within which this study was nested [27].

#### Study participants

Detection and referral of people with probable active convulsive epilepsy whether they were on treatment or not was carried out by community key informants and health extension workers (HEWs) who had been trained for 2 days in case recognition. The diagnosis was then confirmed by PHC workers in the health centres who had been trained in mhGAP-IG. Recruitment into the study took place at the point when the identified person had attended the health centre. After the PHC worker had confirmed the diagnosis of active convulsive epilepsy, they assessed the person and developed a treatment plan in accordance with the mhGAP-IG manual, including prescription of antiepileptic medication and psychotropic medication or psychological support, as indicated regardless of their consent to participate into the study. No diagnostic investigations (e.g. Electroencephalogram EEG) were used to confirm the diagnosis of epilepsy, which is in keeping with mhGAP-IG recommendations, designed to be pragmatic in low-resource settings. A research psychiatric nurse screened potential participants for eligibility, assessed for capacity to consent to participate in the study and obtained informed consent before a person was recruited into the study. This psychiatric nurse did not interact with the PHC workers or provide any assistance with diagnosis.

Eligibility criteria were as follows: i) PHC worker diagnosis of active convulsive epilepsy based on the WHO mhGAP-IG, ii) aged 18 years or above, iii) no plans to out-migrate in the next 12 months, and iv) able to converse in Amharic, the official language of Ethiopia. Participants were excluded if there were communication difficulties due to cognitive or intellectual disability or lack of capacity to consent.

#### Screening instrument

The Self Reporting Questionnaire (SRQ-20)- was developed by the World Health Organization (WHO) to screen for common mental disorder symptoms at primary health care level [28]. The SRQ-20 has been used in various population groups including the elderly and in people with other psychiatric disorders [28]. The instrument has 20 questions with “yes/no” answers and it can be easily administered by an interviewer. Total score on SRQ-20 for a given person ranges from 0 to 20. The 20 items of SRQ-20 cover depressive, anxiety, somatic symptoms and suicidal ideation present in the past 30 days.

The SRQ-20 has been translated into Amharic and has been validated in postnatal women [29] and at primary health care level [30]. It has been culturally adapted and investigated for semantic, content and technical validity in Ethiopia [29, 31] although never in the context of people with epilepsy. Exploratory factor analysis using data collected at a primary health care level in rural Ethiopia produced a single factor structure [30].

#### Standardised reference measure of mental disorders

A semi-structured clinical interview, the Operational Criteria for Research+ (OPCRIT plus) was used as the standardized reference measure of mental disorders [32]. The original OPCRIT consists of an electronic psychopathological checklist of items for diagnosis of psychosis and affective disorders [33]. OPCRIT+ was redeveloped with an expansion of items for additional affective, anxiety, substance use and personality disorders [32]. It also includes items for assessment of risk, psychosocial status and prognosis. The OPCRIT+ provides a simple and reliable method of applying multiple operational diagnostic criteria for diagnosis of a broad range of psychiatric disorders [32]. OPCRIT+ has been used in extensively in the same setting by psychiatry nurses and psychiatrists [34, 35]. It has shown to have good interrater reliability, with a weighted kappa of 0.70 for diagnostic reliability [32].

#### Other measures

*Socio-demographic and epilepsy-related characteristics* were measured using self-report questions for age, gender, educational status, relative wealth, and occupation, types of seizure, seizure frequency / month and duration of epilepsy.

#### Data collection and management

The SRQ-20 was administered by experienced lay data collectors who had completed secondary school education. The OPCRIT+ was administered by psychiatric nurses. The psychiatric nurses had previous training and experience of administering OPCRIT+ [35]. The detection of comorbid mental disorders by PHC workers was evaluated by review of the clinical documentation following consultation. The administration of the SRQ-20 and OPCRIT+ was carried out after the study participants were evaluated by PHC.

To avoid an order effect, the order of administration of the SRQ-20 and the OPCRIT+ assessment was randomised. Both administrators (lay data collectors and psychiatric nurses) were masked to the outcome of the assessment done by the other administrator.

Immediately after the completion of the data collection, the field supervisor checked that all the items of the questionnaire had been completed. All data were kept in a secure cupboard in the project office. Data were anonymised, identifiable through a unique project identification number.

#### Data analysis

The data were double entered using Epi-data version 3.1 and analysed using STATA version 12 [36]. Descriptive statistics using percentages were presented for each of the detection methods (PHC worker, symptom screen and clinical diagnosis). The sensitivity, specificity, positive predictive value (PPV) and negative predictive value (NPV) of PHC diagnosis against the standardised reference of psychiatric nurse diagnosis was calculated.

The total score of the SRQ-20 was used. The Receiver Operating Characteristic curve (ROC) was plotted by including only those people diagnosed to have common mental disorders (depression and anxiety disorders) and taking the psychiatric nurse diagnosis of a mental disorders as a gold standard. The optimal cut off point of SRQ was determined based on the maximum specificity (% of true negatives detected by the SRQ-20) not higher than the sensitivity (% of true positives detected by the SRQ-20). Based on the optimal cut-off score for SRQ-20, the specificity, sensitivity, positive and negative predictive value and Youden’s index (specificity and sensitivity-1) were calculated. The sensitivity and specificity of the combined methods of the PHC diagnosis augmented by the SRQ-20 against the OPCRIT + was also calculated.

Pearson’s chi squared test was used to examine the difference in the reports of each items of SRQ-20 between those with or without depression diagnosed by PHC workers and compared to those diagnosed by psychiatric nurses.

Logistic regression was used to examine the factors which were associated with misdiagnosis of comorbid mental disorder by PHC workers.

## Results

### Sociodemographic characteristics

A total of 246 people with epilepsy (PWE) attended the 11 health centres in the four districts for evaluation. Of these, 237 were eligible and were recruited into the study. Seven people did not fulfil the eligibility criteria and the data of two participants were incomplete. The sociodemographic and clinical characteristics of the study participants are presented in Table 1. All participants were diagnosed with generalised seizures, with a median of one seizure per month. Of the total sample, 89.5% (*n* = 212) had received biomedical treatment previously (at any time) before being recruited to this study.

**Table 1:** Sociodemographic characteristics

Characteristics (Total ***N*** = 237)		Number (%)
**Gender**	Male	140 (59.1)
	Female	97 (40.9)
**Marital status**	Never married	102 (43.1)
	Married	124 (52.3)
	Divorced or widowed	11 (4.6)
**Education**	No formal education	95 (40.1)
	Can read and write	40 (16.9)
	Formal education	102 (43.0)
**Employment**	Employed	21 (8.9)
	Unemployed	15 (6.3)
	Farmer	112 (47.3)
	Housewife	61 (25.7)
	Others^a^	28 (11.8)
**Relative wealth**	Low or very low	169 (71.3)
	Average and above	68 (28.7)
**Area of residence**	Rural	208 (87.8)
	Urban	29 (12.2)
**Religion**	Orthodox Christian	208 (87.8)
	Protestant	15 (6.3)
	Muslim	7 (2.4)
**Age (years)**	Median (IQR)	30 (22–42)
**Duration of epilepsy (years)**	Median (IQR)	11 (0–40)
**Seizure frequency /month**	Median (IQR)	1 (0–2)

*IQR* Interquartile Range, *SD* standard deviation

^a^includes students and other jobs

### Standardised reference diagnosis of mental disorder

The prevalence of mental disorders according to the OPCRIT+ was 13.9% (95% confidence interval (CI) 9.6, 18.2%). Major depressive disorder (MDD) was the most common comorbid disorder (7.2%, *n* = 17), followed by alcohol use disorder (AUD) (2.5%, *n* = 6), then psychosis (2.1%, *n* = 5). One individual (0.4%) was diagnosed as having both MDD and AUD (0.4%). The prevalence of dysthymia was 0.8% (*n* = 2) and that of bipolar disorder was 0.8% (*n* = 2). An equal number of males (10) and females (10) were diagnosed with depression and dysthymia by the psychiatric nurse using OPCRIT+.

### PHC worker diagnosis of mental disorder

Based on the chart review of study participants, 6.3% (95%CI 3.2, 9.4%) of the people with epilepsy were diagnosed by PHC workers as having comorbid mental disorders: 3.4% (*n* = 8) with MDD, 1.7% (*n* = 4) with psychosis and 1.3% (*n* = 3) with AUD. Of the 8 people with a diagnosis of depression by PHC workers, six were males. The sensitivity and specificity of PHC diagnosis was 21.1 and 96.1%, respectively, compared to the standardised reference diagnosis. The positive predictive value (PPV) of PHC worker diagnosis was 46.7% and negative predictive value (NPV) of 88.3%.

### SRQ-20

The total SRQ-20 score was positively skewed. The median score was 7 with IQR of 3–12. When the SRQ-20 score was compared with the standardised reference diagnosis, the optimum cut-off score of SRQ-20 indicating common mental disorder was greater or equal to 9. The area under the ROC of SRQ-20 was 0.74 with 95% confidence interval (CI) 0.62 to 0.82. At this cut-off, 62.1% of participants were classified correctly, although the PPV was very low (15.1%) (See Table 2). The prevalence of common mental disorder at this cut-off (9 or above) was 41.5%.

**Table 2:** Optimal SRQ-20 cut-off for detection of common mental disorder and associated validity coefficients

SRQ-20 Cut off	Prevalence of common mental disorder	Sensitivity (%)	Specificity (%)	Positive predictive value	Negative predictive value	% correctly classified	Youden’s index
≥7	49.6%	90.0	54.4	16.2	98.2	57.6	0.44
≥8	46.0%	75.0	56.9	14.6	95.9	58.5	0.32
≥9	41.5%	70.0	61.3	15.1	95.4	62.1	0.31
≥10	36.2%	65.0	66.8	16.0	95.1	66.5	0.32

Out of the 15 individuals diagnosed to have comorbid mental disorders by PHC workers, only 6 (40%) had an SRQ-20 score of 9 or above.

In people with a PHC worker diagnosis of depression, the most frequently endorsed SRQ-20 items were loss of interest, feeling frightened and getting easily tired. However, there was no significant differences in the reporting of these symptoms between those with or without a PHC worker depression diagnosis. There were multiple depressive and somatic symptoms which discriminated between those with and without standardised reference depression diagnosis by psychiatric nurses (see Table 3).

**Table 3:** Prevalence of each SRQ-20 items in depression diagnostic categories of PHC versus standardised reference diagnosis

Total SRQ-20 score	Comorbid mental disorders in PWE diagnosed by PHC n (%)			Comorbid mental disorders in PWE diagnosed by psychiatric nurses’ n (%)		
	No comorbidity (***n*** = 222)	Depression (***n*** = 8)	Fisher’s exact test (***p*** value)	No comorbidity (***n*** = 204)	Depression (***n*** = 20)	Chi square test (***p*** value)
**Poor sleep**	80 (36)	4 (50)	0.47	70 (34.3)	11 (61.1)	3.38 (0.07)
**Easily frightened**	122 (55)	5 (62.5)	0.73	111 (54.4)	13 (72.2)	0.83 (0.36)
**Hands shake**	93 (41.9)	1 (12.5)	0.15	79 (38.7)	10 (55.6)	0.97 (0.33)
**Worried**	119 (53.6)	4 (50)	1.00	108 (52.9)	12 (66.7)	0.36 (0.55)
**Poor digestion**	55 (24.8)	2 (25)	1.00	44 (21.6)	10 (55.6)	8.05 (0.01)
**Trouble thinking clearly**	81 (36.5)	3 (37.5)	1.00	70 (34.3)	13 (72.2)	7.35 (0.01)
**Feeling unhappy**	78 (35.1)	4 (50)	0.46	65 (31.9)	13 (72.2)	8.81 (< 0.01)
**Crying more than usual**	56 (25.2)	2 (25)	1.00	47(23.0)	11 (61,1)	4.68 (0.03)
**Difficulty to enjoy activities**	69 (31.1)	4 (50)	0.27	62 (30.4)	10 (55.6)	3.21 (0.07)
**Difficulty to make decisions**	78 (35.1)	5 (62.5)	0.14	70 (34.3)	13 (72.2)	7.35 (0.01)
**Daily work suffering**	80 (36)	4 (50)	0.47	71 (34.8)	12 (66.7)	4.96 (0.03)
**Unable to play useful part in life**	87 (39.2)	3 (37.5)	1.00	71 (34.8)	16 (88.9)	15.7 (0.00)
**Loss of interest**	79 (35.6)	5 (62.5)	0.15	67(32.8)	11 (61.1)	3.9 (0.05)
**Feeling worthless**	89 (40.1)	4 (50)	0.72	77 (37.7)	12(66.7)	3.8 (0.05)
**Thoughts of ending life**	43 (19.4)	0	0.36	33 (16.2)	9 (50.0)	9.93 (0.00)
**Feel tired**	132 (59.5)	5 (62.5)	1.00	118(57.8)	16 (88.9)	3.72 (0.05)
**Easily tired**	100 (45.1)	4 (50)	1.00	84 (41.2)	16 (88.9)	11.1 (0.00)
**Poor appetite**	91 (41)	3 (37.5)	1.00	78 (38.2)	12 (66.7)	3.59 (0.06)
**Uncomfortable stomach**	56 (25.2)	1 (12.5)	0.68	43 (21.1)	10 (55.6)	11.1 (0.004)
**Frequent headaches**	137 (61.7)	3 (37.5)	0.16	118 (57.8)	17 (85.0)	5.61 (0.02)

The combination of both diagnostic methods (the SRQ-20 score above the optimum cut off augmented by PHC diagnosis of depression) was also compared with the standardised reference diagnosis of depression. The sensitivity of this combined approach was 78.9% (95% confidence interval (CI) 73.4, 84.4%) with specificity of 59.7% (95% CI 53.2, 66.2%). However, the PPV was low, at 15.6%.

Factors associated with misdiagnosis of comorbid mental disorders by PHC workers were also examined. As shown in Table 4, only age was significantly associated with misdiagnosis of comorbid mental disorders by PHC workers (adjusted odds ratio (OR) 1.06, 1.02 1.11 for every increasing year of age).

**Table 4:** Sociodemographic and epilepsy related factors associated with missed diagnosis of comorbid mental disorders by PHC workers

Characteristics	Univariate analysis		Multivariable analysis	
	Crude odds ratio (c OR)	95% CI	Adjusted Odds Ratio (aOR)	95% CI
**Age (years)**	1.03	1.00, 1.06	1.06	1.02 1.11
**Gender**				
**Male**	1		1	
**Female**	1.27	0.56, 2.88	1.67	0.69, 4.09
**Relative wealth**				
**Average and above**	1		1	
**Very low or low**	1.10	0.44, 2.76	1.42	0.53, 3.78
**Education**				
**No formal**	1		1	
**Formal education**	0.97	0.42, 2.21	1.16	0.46, 2.95
**Marital status**				
**Never married or formerly married**	1		1	
**Married**	1.09	0.48, 2.46	2.22	0.82, 6.06
**Residency**				
**Urban**	1		1	
**Rural**	1.08	0.30, 3.84	0.96	0.25, 3.67
**Seizure frequency**	1.04	0.96, 1.13	1.06	0.97, 1.15
**Duration epilepsy (years)**	1.00	0.96, 1.04	10.97	0.93, 1.02

## Discussion

In this cross-sectional survey, the performance of primary health care (PHC) workers in diagnosing comorbid mental disorders against a standardised measure and a screening instrument (SRQ-20) for common mental disorders was examined. The sensitivity of the PHC workers’ diagnoses was low, although they had high specificity in relation to the standardised reference diagnosis. The psychometric properties of SRQ-20 indicate an optimal cut-off score of 9 and above, with moderate sensitivity and specificity but low positive predictive value. When the two diagnostic methods (SRQ-20 screening augmented by PHC workers diagnosis) were combined, the sensitivity was markedly improved but the positive predictive value remained low. Misdiagnosis of comorbidity by PHC worker was significantly associated with increasing age.

The low sensitivity of the PHC workers in detection of depression in our study sample is consistent with other studies carried out in Ethiopia and other parts of the world [18, 37–39]. In this current study, less than half (45.5%) of the people with epilepsy and comorbid mental disorders were detected by PHC. Very little research attention has been paid to investigating the detection of comorbid mental disorders in people with epilepsy in routine clinical practice in low-income country settings [22]. Under detection and management of comorbid common mental disorders has consistently been found to be a problem in high income countries (HIC), despite the high prevalence of co-morbidity in people with epilepsy [7, 40]. One of the reasons identified for this under-detection is the soloed approach to care for people with epilepsy and mental health conditions, both in terms of inadequate training of neurologists in the psychiatric aspects of mental disorders and poor communication between the neurologist and psychiatrist [40]. In addition, failure of the training programme on psychiatric aspects of commonly occurring neurologic disorders for psychiatry residents, lack of interest in neurologic literature and the absence of psychiatrist in the team of neurologists were seen in a study from the USA [40].

This issue in low income countries like Ethiopia is different from the HIC where more frontline management of epilepsy is expected to be carried out in primary care, with little access to either neurologists or psychiatrists [24]. Health professionals working at the primary health care level are also expected to detect and manage five priority mental disorders based on their training through mhGAP [24]. It was shown previously that the PHC workers in rural primary health care in Ethiopia were more likely to detect people who presented with psychological than somatic symptoms, even though somatic symptoms are the more common presenting symptoms, and tended to detect those who had more severe forms of depression [18]. There were also multiple system and individual level barriers for under detection of depression by PHC workers. Some of these barriers included poor training of the PHC workers on mental disorders, non-biomedical explanatory models of mental disorders by patients and their family members, and somatic symptoms being the common presenting complaints of the patients [18]. In people with epilepsy, overlaps between symptoms of depression and the side effects of older classes of antiepileptic medications (e.g. phenobarbitone, phenytoin) makes early detection of depression challenging [41].

The sensitivity of SRQ-20 in screening for depression was not at a satisfactory level in people with epilepsy but the scale performed relatively well in the general population of Ethiopia and in a similar setting of Eritrea [30, 42, 43]. There is minimal evidence on the validation of SRQ-20 in special populations like people with epilepsy which has made it difficult to compare our findings to previous work. Systematic reviews of validation studies of screening instruments for common mental disorders in LMICs in the general population have shown that the instruments with best performance are those which have been locally developed from scratch for specific population in a specific setting [44]. The SRQ-20 is unusual because it was developed with LMIC contexts in mind, drawing on symptoms from scales used in both HIC and LMIC settings [28]. In the previous studies of SRQ-20 in Ethiopia, the local adaptation and attempts to make the instrument culturally sensitive are likely to have contributed to the observed validity [42]. However, in people with epilepsy, the high number of somatic items on the SRQ-20 may overlap with impacts of inadequately controlled epilepsy (e.g. on sleep) and the effects of antiepileptic drug side effects [45].

Less than half of the participants diagnosed by the PHC workers as having comorbid mental disorders scored above the optimum cut off point of SRQ-20. Specific items of the SRQ-20 were not differentially reported by the participants diagnosed to have comorbid depression by the PHC workers. This could be due to the small number of participants diagnosed to have comorbid depression by the PHC workers. Some of the somatic and depressive symptom items of SRQ-20 were also highly prevalent in both population (with or without depression). It has been previously shown in the same setting that people with depression spontaneously reported symptoms which are partially represented in the typical diagnostic criteria [46]. The concept of depression was not well recognized as an illness in this community unless it is usually associated with disruption of function [46]. Since the PHC workers working in rural settings are usually accustomed to the cultural ways of expressing distress, this could be inconsistent with the symptoms of depression represented in SRQ-20 and reduce the sensitivity.

This study has also demonstrated that use of the two methods (SRQ-20 screening augmented by the clinical evaluation) of detection comorbidity of depression is promising. This augmentation is seen by the high specificity of PHC workers which has compensated for the low specificity of SRQ-20. As people with epilepsy are a high risk population, the routine use of depression screening is highly recommended in HIC [7]. It is also recommended that the ideal screening tool should be able to detect depression even when patients are presenting with somatic complaints [47]. PHC workers knowing the common terms patients use for emotional symptoms and their relevance will help in identification of mental disorders [47]. However, in our study, the screening questionnaire was administered by research staff and was not used as part of clinical decision-making. Implementation of screening questionnaires for depression in routine PHC settings has yielded mixed results in high-income countries [48], indicating the need for future studies to evaluate the effectiveness of introduction of routine screening in people with epilepsy in LLMICs.

The inclusion and evaluation of three different diagnostic methods simultaneously in routine clinical care was one of the strengths of this study. It is also one of the few studies carried out on detection of comorbid mental disorders in people with epilepsy in sub-Saharan Africa. The results of this study indicate that attention needs to be focused on improving the detection of co-morbid mental health problems integrated within the scale-up of primary care-based mental health care in Ethiopia and similar settings. This study was a pragmatic study which has followed mhGAP-IG criterion for evaluation of people with epilepsy. The use of psychiatric nurses for the standardised clinical assessment rather than psychiatrists means that it may not be considered a gold standard. However, in the Ethiopian setting, psychiatric nurses take on additional clinical roles compared to their counterparts in high-income countries, including diagnostic assessment, and would have had experience and competence in this area. Even though OPCRIT+ allows local clinicians to probe and explore responses in a flexible way, it has not been validated in this specific context. The other limitation of this study was the absence of people with non-generalized seizures. This is likely to be because of the limited clinical experience of PHC workers in management of mental or neurological disorders before the implementation of mhGAP. Even after the mhGAP training the diagnosis of focal seizures and non-epileptic seizures needs experience and an EEG. Neither the primary health care facility nor the PHC workers are equipped with this skill or resource. The low detection of anxiety disorders in this study could also be due to the presentation of depression in Ethiopian culture, which tends to be a combination of anxiety, somatic and depressive symptoms rather than typical DSM criteria, alongside the non-biomedical causal attributions of depressive/anxiety symptoms in this society [27]. It is possible that psychiatry nurses tended to prioritise a diagnosis of depression over anxiety disorders as this reflected their experince of presentation in clinical settings.

Further, studies are needed on interventions to increase detection. The development, cultural adaptation and evaluation of the impact of symptom screening tools in routine settings is a promising avenue for future research. Training programmes for PHC workers in LMICs, such as mhGAP, may benefit from a more horizontally integrated diagnostic algorithms which facilitate detection of co-morbidity. Contextualisation using local expression of mental distress may also increase the impact on detection.

## Conclusion

In conclusion the detection of co-morbid mental disorders in people with epilepsy by PHC workers was low. The use of screening instruments augmented by the clinical skill of the PHC workers may possibly improve the detection of mental disorders but needs further evaluation.

## Supplementary Information

**Additional file 1: Data set.** Performance of primary health care workers in detection of mental disorders comorbid with epilepsy in rural Ethiopia.

## Data Availability

All data generated or analysed during this study are included in this published article as an Additional file 1- Data set.

## Abbreviations

HIC: High-income countries
ADHD: Attention deficit hyperactivity disorder
LLMICs: Low- and lower middle-income countries
WHO: World Health Organization
MNS: Mental neurological and substance use disorders
mhGAP-IG: Mental health Gap Action Programme Intervention Guide
PHC: Primary health care
SNNPR: Southern Nations Nationalities and Peoples’ region
PRIME: PRogramme for Improving Mental health CarE
HEW: Health extension workers
EEG: Electroencephalogram
SRQ-20: Self Reporting Questionnaire
OPCRIT+: Operational Criteria for Research
PPV: Positive predictive value
NPV: Negative predictive value
ROC: Receiver Operating Characteristic curve
PWE: People with epilepsy
MDD: Major depressive disorder
AUD: Alcohol use disorder
